# Increased Dickkopf-1 expression in breast cancer bone metastases

**DOI:** 10.1038/sj.bjc.6603959

**Published:** 2007-09-18

**Authors:** N Voorzanger-Rousselot, D Goehrig, F Journe, V Doriath, J J Body, P Clézardin, P Garnero

**Affiliations:** 1 Molecular Markers, Synarc, Lyon F-69416, France; 2 INSERM, Research Unit 664, Lyon F-69372, France; 3 Université Claude Bernard Lyon 1, Villeurbanne F-69622, France; 4 Laboratory of Endocrinology & Bone Diseases and Department of Medicine, Institut Jules Bordet, University Libre de Bruxelles, Brussels, Belgium

**Keywords:** Wnt signalling, Dickkopf-1, breast cancer, bone metastasis, bone turnover

## Abstract

The aim of this study was to determine whether Dickkopf-1 (Dkk-1) expression in breast cancer was associated with bone metastases. We first analysed Dkk-1 expression by human breast cancer cell lines that induce osteolytic or osteoblastic lesions in animals. Dickkopf-1 levels were then measured in the bone marrow aspirates of hind limbs from eight NMRI mice inoculated with breast cancer cells that induced bone metastases and 11 age-matched non-inoculated control animals. Finally, Dkk-1 was measured in the serum of 17 women with breast cancer in complete remission, 19 women with breast cancer and bone metastases, 16 women with breast cancer and metastases at non-bone sites and 16 healthy women. Only breast cancer cells that induce osteolytic lesions in animals produced Dkk-1. There was a six-fold increase in Dkk-1 levels in the bone marrow from animals inoculated with MDA-B02 cells when compared with that of control non-inoculated animals (*P*=0.003). Median Dkk-1 levels in the serum of patients with breast cancer and bone metastases were significantly higher than levels of patients in complete remission (*P*=0.016), patients with breast cancer having metastases at non-bone sites (*P*<0.0001) and healthy women (*P*=0.047), although there was a large overlap in individual levels between the different groups. In conclusion, Dkk-1 is secreted by osteolytic human breast cancer cells lines and increased circulating levels are associated with the presence of bone metastases in patients with breast cancer. Measurements of circulating Dkk-1 levels may be useful for the clinical investigation of patients with breast cancer and bone metastases.

One of the main complications of breast cancer is bone metastases (Mundy, 2002; Body, 2006a). Although bone metastases from breast cancer are more often characterised by osteolytic lesions, osteoblastic or mixed lesions may also occur. Breast cancer cells secrete different factors (parathyroid hormone-related protein, interleukins 6, 8, and 11, and prostaglandins) that stimulate the activity of osteoclasts (bone-lysing cells), leading to the subsequent degradation of bone (Guise *et al*, 2005; Yin *et al*, 2005). Breast cancer-derived endothelin-1, as a stimulator of the activity of osteoblasts (bone-forming cells), is probably the main mediator of osteoblastic lesions (Guise *et al*, 2005). Endothelin-1 also inhibits osteoclast activity (Guise *et al*, 2005), indicating that the formation of osteoblastic lesions is the result of stimulation of bone formation and inhibition of bone resorption by breast cancer cells. It is most likely that the formation of breast cancer osteolytic lesions reflects stimulation of osteoclasts and inhibition of osteoblasts. Breast cancer-derived factors involved in the inhibition of osteoblast activity are however unknown.

It has been recently shown that the Wnt signalling pathway plays a pivotal role in the differentiation and activity of osteoblastic cells (Day *et al*, 2005). There are 19 closely related Wnt genes that have been identified in humans (Katoh, 2002). The primary receptors of Wnt molecules are the seven-transmembrane Frizzled proteins (FRP), each of which interact with a single transmembrane low-density lipid (LDL) receptor-related protein 5/6 (LRP5/6) (He *et al*, 2004). Different secreted proteins, including soluble FRP-related proteins, Wnt inhibitory factor-1 (WIF1), and Dickkopfs (Dkk) 1–4 prevent ligand–receptor interactions and consequently inhibit the Wnt signalling pathway (Kawano and Kypta, 2003). Wnt proteins activate several intracellular signalling pathways through either a ‘canonical’ or a ‘non-canonical’ pathway (Pandur *et al*, 2002). In the canonical pathway, secreted Wnt ligands bind to FRP proteins and regulate the stability of *β*-catenin (Nusse, 2005). In the absence of a Wnt signal, *β*-catenin is constitutively downregulated by a multicomponent destruction complex (Aberle *et al*, 1997; Hart *et al*, 1998). In the presence of secreted Wnt, this degradative process is inhibited, leading to the accumulation of *β*-catenin in the nucleus and regulation of Wnt-responsive genes (Eastman and Grosschedl, 1999).

Among the different inhibitors of the canonical Wnt signalling pathway, genetic disorders indicate that Dkk-1 plays a major role in the regulation of bone metabolism. For example, in the syndrome of hereditary high bone density (Boyden *et al*, 2002), mutations in the LRP5 gene prevent binding of Dkk-1 to LRP5 and stimulate growth and differentiation of osteoblasts, leading to increased bone mass. More recently, it has been shown that Wnt signalling pathways and Dkk-1 may also play an important role in malignant bone diseases (Tian *et al*, 2003; Hall *et al*, 2005). Tian *et al* (2003) have shown that increased Dkk-1 levels in the plasma from bone marrow aspirates of patients with multiple myeloma are associated with the presence of osteolytic lesions. In addition, Dkk-1 produced by myeloma cells inhibits osteoblast differentiation *in vitro* (Tian *et al*, 2003). Hall *et al* (2005) have shown that the inhibition of Dkk-1 expression in osteolytic PC-3 prostate cancer cells induced *in vitro* osteoblastic activity in particular phosphatase alkaline activity and mineralisation in murine bone marrow stromal cells. Conversely, the induction of Dkk-1 in the mixed osteoblastic/osteolytic prostate cancer C4-2B cell line results in experimental bone metastases with an osteolytic phenotype *in vivo*. Dickkopf-1 expression in human breast cancer cell lines has been recently reported at mRNA level (Schwaninger *et al*, 2007).

The aim of this study was to determine whether Dkk-1 expression in breast cancer was associated with the presence of bone metastases.

## MATERIALS AND METHODS

### ELISA for Dkk-1

**Figure 1:**
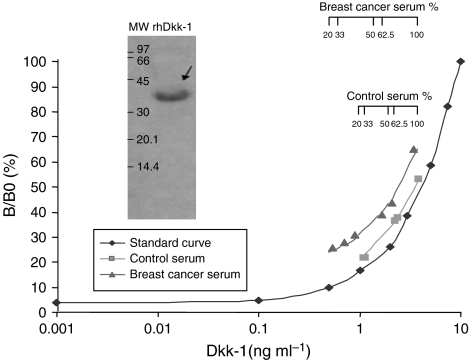
Typical standard curve of the Dickkopf-1 (Dkk-1) ELISA was obtained by making serial dilutions of recombinant human Dkk-1 (rhDkk-1) of known concentrations used as a standard (*x* axis). The *y* axis shows the optical density (OD) obtained for each concentration of the standard (B) expressed as a percentage of the OD value observed with the highest concentration of Dkk-1 (B0). Dose–dilution curves of serum samples from a healthy post-menopausal woman and from a post-menopausal woman with breast cancer and bone metastases were parallel to the standard curve. Inset: Western blot indicating that the polyclonal antibody used in the ELISA recognises human Dkk-1 at the expected 37 kDa mass (arrow), mw=molecular weight markers.

Microtitre plates (Nunc ImmunoMaxiSorp, Dutscher, Issy-Les-Moulineaux, France) were coated with an anti-human Dkk-1 antibody produced in goats immunised with purified, insect cell line *Sf* 21-derived, recombinant human Dkk-1 (rhDkk-1) (R&D System, Minneapolis, MN, USA), diluted in phosphate-buffered saline (PBS), pH 7.4 (137 mM NaCl, 3.3 mM KCl, 8 mM Na_2_HPO_4_, 1.47 mM KH_2_PO_4_, pH 7.4) and incubated for 2 h at room temperature. Plates were then incubated for 2 h at room temperature with PBS, and 4% BSA (Euromedex, Souffelweyersheim, France). After five cycles of washing with PBS (pH 7.4), 1% BSA, and 0.1% Tween 20 (washing buffer), rhDkk-1 used as standard (R&D System), culture supernatant, bone marrow aspirate or serum were incubated for 2 h at room temperature. After washing, the biotinylated form of the anti-human Dkk-1 antibody was added for 1 h at room temperature. Streptavidin–horseradish peroxidase (Jackson ImmunoResearch Laboratories, Cambridgeshire, UK) was then added with the substrate solution of 3,3′,5,5′-tetramethylbenzidine (TMB) (Euromedex). Finally, the reaction was stopped by the addition of 100 *μ*l of 0.2 M H_2_SO_4_. The optical density (OD), which is proportional to the concentration of Dkk-1 was measured at a wavelength of 450 nm corrected for the absorbance at 650 nm. The polyclonal antibodies for Dkk-1 used in the assay have no significant crossreactivity for human or murine Dkk-4 and but crossreact at 85% with recombinant murine Dkk-1. We performed a full technical validation of the ELISA. The detection limit, defined as the concentration corresponding to 2 s.d. above the mean of 25 determinations of the zero calibrator, was determined to be 0.25 ng ml^−1^. The intra-assay variation assessed by 20 measurements of two different serum samples (mean levels of 13.8 and 42.2 ng ml^−1^) in the same run were 9.0 and 5.9%, respectively. The interassay variation determined by the measurements of two different serum samples (mean levels of 11.9 and 18.1 ng ml^−1^) in 10 different runs were 14.8–13.6%, respectively. A typical standard curve is shown in Figure 1. The median dilution recovery of serum was 95% and the dose–dilution (from 20 to 62.5%) curves of two serum samples, one from a healthy subject and one from a breast cancer patient with bone metastases, were parallel to the standard curve (Figure 1). Western blot analysis showed that the antibody used in ELISA test recognises recombinant human Dkk-1 protein at the expected 35 kDa band (Figure 1 inset).

### Western blot analysis for Dkk-1

RhDkk-1 (0.1 *μ*g ml^−1^, 10 *μ*l) was heated 5 min at 100°C in loading buffer (4% SDS, 200 mM DTT, 100 mM Tris, pH 6.8, 20% glycerol, 0.1% bromophenol blue). Denatured samples were run on a 12% acrylamide gel. After transfer the PVDF membranes were blocked overnight in TBS, pH 7.2, 3% milk, and 0.1% Tween 20. The membranes were then incubated with the anti-human Dkk-1 antibody (R&D System) at a dilution of 1 : 1000, washed and incubated with peroxidase-conjugated rabbit anti-goat secondary antibody (Jackson ImmunoResearch Laboratories/Beckman Coulter, Roissy, France) at a dilution of 1 : 10 000. The reaction was revealed using the luminol-based enhanced chemiluminescence detection system (ECL Amersham Biosciences, Saclay, France).

### Cell lines and culture conditions

Human breast cancer cell lines (MDA-MB-231, MCF-7, ZR-75, and T47D) were obtained from the American Type Culture Collection (Rockville, MD, USA). The bone features of these cells lines have been described previously as predominantly osteolytic for MDA-MB-231, MCF-7 and predominantly osteoblastic for ZR-75 and T47D (Evans *et al*, 1991; Hullinger *et al*, 2000; Guise *et al*, 2003; Schwaninger *et al*, 2007). MDA-MB-231/B02, also named MDA-B02, is a subclone of the primary tumour cell line MDA-MB-231, which has been selected for its unique predilection to metastasise to bone when injected into the tail vein of nude mice as described previously (Peyruchaud *et al*, 2001).

Breast cancer cells were grown in DMEM (Life Technologies, Gibco BRL, Cergy Pontoise, France) supplemented with 10% fetal calf serum (FCS) (Bio-Media, Boussens, France), 1% (v v^−1^) penicillin/streptomycin and 2 mM L-glutamine (Life Technologies) at 37°C in a 5% CO_2_ incubator until confluence. Cells were then cultured for 24 h in the medium described above but without FCS. The supernatant was collected, centrifuged for 10 min at 1000 r.p.m., and stored frozen at −70°C until analysis for Dkk-1.

### Reverse transcription and real-time quantitative PCR analysis of Dkk-1

Total RNA from breast cancer cell lines was extracted using the Total RNA Isolation System (Promega, Charbonnières, France). cDNA was then obtained using the Moloney murine leukaemia virus-1 reverse transcriptase (Promega). The following primers previously described were used for amplification of human Dkk-1 gene: 5′-TAGCACCTTGGATGGGTATT-3′ (sense) and 5′-ATCCTGAGGCACAGTCTGAT-3′ (antisense) generating a PCR product of 110 bp (16). GAPDH mRNA expression was analysed in parallel to control the amount of cDNAs in each reaction. The following primers were used for amplification of GAPDH gene: 5′-CCTGGCCAAGGTCATCCATGACAAC-3′ (sense) and 5′-CAGTTCGAGTAAAGGACCATACTGT-3′ (antisense). PCR were run using a program consisting of 30 cycles of denaturation at 94°C for 1 min, annealing at 58°C for 1 min, and extension at 72°C for 1 min with a preincubation of 94°C for 2 min. Products from RT–PCR were separated by electrophoresis on a 2% agarose gel and then visualised with ethidium bromide under ultraviolet light. Human Dkk-1 mRNA was quantified by real-time PCR using the QuantiFast SYBR Green PCR kit (Qiagen S.A., Courtaboeuf, France). Fluorescence was monitored and analysed in a Light Cycler (Roche Diagnostics, Meylan, France). GAPDH mRNA expression was analysed in parallel to confirm the use of equal amount of cDNAs in each reaction and all quantitations were normalised to this endogenous control. The relative quantitation value of Dkk-1 mRNA compared to that of GAPDH control gene was expressed as 2^−(*C*_Dkk-1-_*C*_GAPDH_)^ where *C*_Dkk-1_ and *C*_GAPDH_ are the mean threshold cycle of the PCRs in triplicate for Dkk-1 and GAPDH, respectively (Yang *et al*, 2004).

### Mice model of breast cancer-induced bone metastases

Female NMRI nude mice of 4 weeks of age (Elevage Janvier, Le Genest-St-Isle, France) were used. Housing and care, method of euthanasia, and experimental protocols, were conducted in accordance with a code of practice established by the Experimentation Review Board from the Laennec School of Medicine, Lyon, France.

MDA-B02 cells (5 × 10^5^ cells in 100 *μ*l of PBS) were inoculated into the tail vein of anaesthetised mice. Thirty days after tumour cell inoculation, animals having radiographic evidence of osteolytic lesions were killed. In all B02 inoculated mice both tibias presented with bone tumour lesions. Consequently, for each animal, the bone marrow of both tibias was flushed with 200 *μ*l of DMEM and mixed before analysis for Dkk-1 levels. Similar experiments were conducted with age-matched control mice that had not been inoculated with MDA-B02 cells. Bone marrow was then centrifuged and the supernatant was stored frozen at −70°C until ELISA for Dkk-1.

*Radiographs* Whole body radiographs (MIN-R2000 films; Kodak, Rochester, NY, USA) of anaesthesised animals were taken 30 days after tumour cell inoculation using an MX-20 cabinet X-ray system (Faxitron X-ray Corporation, Wheeling, IL, USA). The area of osteolytic lesions of the whole body was measured using a Morpho computerised image analysis system (Altavista, France), and the extent of bone destruction per animal was expressed in square millimetres, as described previously (Peyruchaud *et al*, 2003).

*Bone histology* Hind limbs from animals were fixed and embedded in methylmethacrylate. Seven-micrometre sections of undecalcified long bones were stained with Goldner's trichrome. Histological analyses were performed on longitudinal medial sections of tibial metaphases using a Morpho computerised image analysis system (Altavista, France), as described previously (Peyruchaud *et al*, 2003).

### Circulating Dkk-1 in women with breast cancer

Fasting serum samples from 52 women with breast cancer consecutively recruited at the Department of Medicine in Institut Jules Bordet in Brussels were analysed. All serum samples were kept frozen at −70°C until the assay. All patients had a histologically confirmed breast cancer. Among the 52 women with breast cancer, 17 had breast cancer in complete remission, 19 had radiological evidence of bone metastases only, and 16 had metastases at non-bone sites (hepatic, cutaneous, lymph node, and lung) (Table 1). Patients were on stable antineoplastic therapy for at least 4 weeks and did not receive previous treatment with bisphosphonates within the previous 4 months. Patients did not have a history of other metabolic bone disease. Serum Dkk-1 levels was also determined in 16 healthy untreated women (median age 50 years; range: 39–60 years) recruited from a blood donor program with no history of breast disease or metabolic bone disease.

### Biochemical markers of bone formation and bone resorption

Serum bone alkaline phosphatase (bone ALP) – a marker of bone formation – was measured by an immunochemiluminescence assay using the Ostase reagent on an automatic analyzer (Ostase, Access, Beckman Coulter, Fullerton, CA, USA). The intra-assay and interassay CV are less than 3.5 and 8%, respectively. The crossreactivity of the assay with the liver isoenzyme is of 13%.

Serum C-terminal crosslinking telopeptide of type I collagen (S-CTX) was measured by a two-site assay using monoclonal antibodies raised against an eight amino-acid sequence from the C-telopeptide of human type I collagen by an automatic analyser (Elecsys, Roche Diagnostic, Mannheim, Germany). Intra-assay variation is lower than 3% and interassay variation is lower than 5%.

### Statistical analysis

Statistical analyses were performed using the Statistical Analysis System 8e (SAS Institute Inc., Cary, NC, USA). Differences of bone marrow Dkk-1 levels between groups of mice were assessed by the non-parametric Mann–Whitney test. Because serum Dkk-1 levels were not normally distributed, data were log transformed to achieve normal distribution before analyses. Differences of serum Dkk-1 levels between groups of patients were assessed by ANOVA test. *P*-values less than 0.05 were considered statistically significant.

## RESULTS

### Dickkopf-1 is expressed and secreted by human breast cancer cell lines that induce osteolytic bone lesions in animals

**Figure 2:**
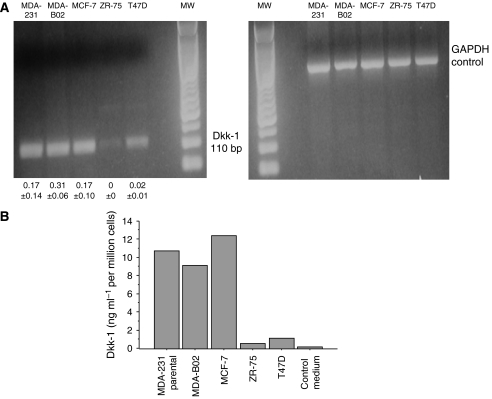
(**A**) RT–PCR of Dickkopf-1 (Dkk-1) and GAPDH for the breast cancer cell lines MDA-231, MDA-B02, MCF-7, ZR-75, and T47D. RT–PCR fragments were separated on a 2% agarose gel and then stained with ethidium bromide. Numbers below the first panel correspond to the relative levels of Dkk-1 mRNA measured by real-time PCR expressed as the mean±s.d. of the PCRs in triplicate in each cell line. (**B**) Dkk-1 levels by ELISA in the supernatant of breast cancer cell lines.

The expression of Dkk-1 in human breast cancer cell lines was analysed by RT–PCR. Agarose gel electrophoresis of RT–PCR products showed a single correctly sized band corresponding to Dkk-1 mRNA for each positive sample (Figure 2A). The integrity and similar relative amounts of mRNAs between conditions were confirmed using GAPDH as a constitutively expressed marker (Figure 2A). Real-time RT–PCR Dkk-1 showed significant levels of expression of Dkk-1 mRNA by MDA-MB-231, MDA B02, and MCF-7 cell lines. In contrast, the ZR-75 cell line did not express Dkk-1 and very low mRNA expression was detected in T47D cells (Figure 2A).

The secretion of Dkk-1 was assessed by ELISA of the culture supernatant of human breast cancer cell lines. In agreement with RT–PCR findings, high concentration of Dkk-1 was measured in the culture supernatants of MDA-MB-231, MDA-B02, and MCF-7 cell lines but not in that of ZR-75 and T47D (Figure 2B).

### Dickkopf-1 is present in the bone marrow of bone metastases bearing limbs from MDA-B02-inoculated mice

**Figure 3:**
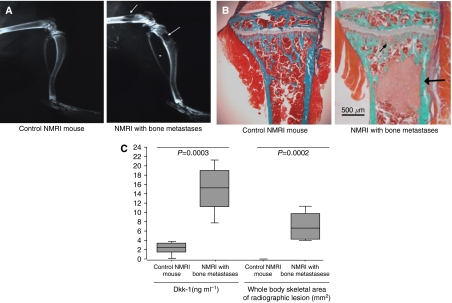
(**A**) Radiographs from hind limbs of non-inoculated control NMRI mouse and osteolytic bone lesions from female nude mice intravenously inoculated with MDA-B02 cells. Whole body radiographs were taken 30 days after tumour inoculation with cells. Osteolytic lesions are indicated by arrows. (**B**) Representative bone histology of Goldner's trichrome-stained tibial metaphysis from control and metastatic animals. Bone is stained in green; bone marrow and tumour cells are stained red. Trabecular bone was completely destroyed and replaced by tumour cells (arrow) in the tibial metaphysis from metastatic animals. (**C**) Box plots of bone marrow levels of Dickkopf-1 (Dkk-1) and whole body skeletal extent of radiological lesions in bone of control (*n*=11) and MDA-B02 inoculated (*n*=8) NMRI mice. From the bottom up, the box indicates the 25th, 50th (median) and 75th percentiles, while the bars indicate the 10th and 90th percentiles, respectively. Statistical significance levels between mice groups were obtained from non-parametric Mann–Whitney test.

All MDA-B02-inoculated mice presented with skeletal radiological (Figure 3A and C) and histological evidence of bone lesions (Figure 3B) in both tibias. As shown in Figure 3C, the median concentration of Dkk-1 in the bone marrow of tibias of mice inoculated with MDA-B02 was six-fold higher than that of control mice (*P*=0.0003; Figure 3C).

### Serum Dkk-1 levels are increased in women with breast cancer and bone metastases

There was no significant association between serum Dkk-1 and age when all subjects were considered together or in each group separately (data not shown).

**Figure 4:**
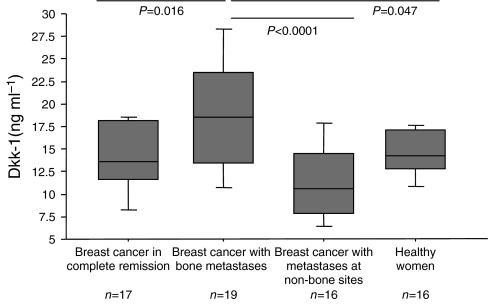
Box plot of serum Dickkopf-1 (Dkk-1) levels in women with breast cancer in complete remission, breast cancer with bone metastases, breast cancer with metastases at non-bone sites, and healthy women. From the bottom up, the box indicates the 25th, 50th (median) and 75th percentiles, while the bars indicate the 10th and 90th percentiles respectively. *P*-values indicate the statistical significance levels of the difference between median serum Dkk-1 levels of patients with bone metastases compared to the other groups.

There was a significant difference in serum Dkk-1 levels between the different groups of patients with breast cancer and healthy controls by ANOVA (*P*=0.0002). As shown in Figure 4, median serum Dkk-1 levels were significantly higher in women with breast cancer and bone metastases compared to women with breast cancer in complete remission (*P*=0.016), women with breast cancer and metastases at non-bone sites (*P*<0.0001) and healthy women (*P*=0.047). There was no significant difference in serum Dkk-1 levels between women with breast cancer in remission, breast cancer at non-bone sites and healthy women. In patients with bone metastases, there was no significant association of serum Dkk-1 with the type of lesions, nor the bulk of the bone metastases (data not shown), although the number of patients in each subgroup was limited.

Serum bone ALP (13.7±6.2 *vs* 9.3±2.1 ng ml^−1^, *P*=0.03 in women with breast cancer and bone metastases and healthy controls, respectively) and serum CTX (0.55±0.56 *vs* 0.22±0.35 ng ml^−1^, *P*=0.0004) were significantly higher in women with breast cancer and bone metastases compared to healthy women. There was no significant association between serum Dkk-1 and serum bone ALP or CTX in women with breast cancer and bone metastases (data not shown).

## DISCUSSION

In this study, we found that Dkk-1 is produced by human osteolytic breast cancer cells *in vitro* and higher serum levels are associated with the presence of bone metastases in patients with breast cancer.

Using real-time quantitative RT–PCR and quantitative ELISA, we demonstrated that Dkk-1 was expressed and secreted in large amount only by cultured human breast cancer cell lines (MCF-7, MDA-MB-231, and MDA-B02) known to induce osteolytic bone metastases in mice. Our findings extend those recently reported by Schwaninger *et al* (2007), showing that Dkk-1 mRNA was expressed in the osteolytic MDA-MB-231 breast cancer line *in vitro*, whereas osteoblastic breast cancer cell lines T47D and ZR-75-1 did not express Dkk-1. Moreover, we found high Dkk-1 protein levels in the bone marrow of tumour-bearing legs from mice inoculated with MDA-B02 breast cancer cells. Our data are in agreement with the observation that PC-3 prostate cancer cells, which induce osteolytic lesions in animals, secrete Dkk-1 and that osteoblastic lesions caused by C4-2B prostate cancer cells revert to an osteolytic phenotype upon transfection of C4-2B cells with a plasmid encoding for Dkk-1 (Hall *et al*, 2005). Because the antibody we used crossreacts with human and murine Dkk-1, we could not discriminate between tumour-derived and bone marrow-derived Dkk-1. Because Dkk-1 could not be detected in the bone marrow of naive animals, this suggests that MDA-B02 tumour cells were the major source of Dkk-1 in the bone marrow. However, because both tibias of mice inoculated with MDA-B02 cells presented with bone metastatic lesions, we could not analyse Dkk-1 levels in non-tumour-bearing legs from inoculated mice, which would have been the optimal control. Additional mechanisms, such as the stimulation by bone-residing breast cancer cells of Dkk-1 expression by bone marrow cells, may also be involved in the high local production of Dkk-1. Such a possibility merits further investigation.

The relevance of our preclinical findings in humans is suggested by the increased levels of Dkk-1 in the serum of patients with breast cancer and bone metastases measured by a new sensitive and reproducible assay that we developed. Although median levels were significantly higher on a group basis in patients with bone metastases than in healthy controls and women with breast cancer and metastases at sites other than bone, there was a large overlap in individual values between the different groups. If confirmed by larger studies, this suggests that the measurement of circulating Dkk-1 would be of limited value to provide an accurate diagnosis of the presence of bone metastases. We found lower but detectable levels of Dkk-1 in women with breast cancer in complete remission and in healthy women. This circulating level probably reflects basal production of Dkk-1 by various normal cells. Interestingly, serum Dkk-1 levels in women with breast cancer and metastases at non-bone sites were lower than values found in patients with bone metastases and were not different from those of healthy women. These data suggest that the secretion of Dkk-1 by cancer cells is increased when present in the bone marrow cavity and/or that cancer cells increased production of Dkk-1 by the bone marrow environment through mechanisms, which are yet unknown. As exemplified with the results obtained in our animal model of breast cancer bone metastasis, one of these potential mechanisms could be related to the stimulatory effect of tumour cells on Dkk-1 production by bone marrow cells since, in the absence of tumour cells, we did not detect Dkk-1 in bone marrow extracts.

Bone-derived RANK-L and several tumour-derived factors including parathyroid hormone related-protein, interleukins 6, 8, and 11, and prostaglandins are involved in the development of breast cancer osteolytic lesions by promoting both osteoclastogenesis and osteoclast activity (Guise *et al*, 2005; Yin *et al*, 2005). However, it is also possible that osteolytic breast cancer cells not only secrete pro-osteoclastic factors, but also inhibitors of osteoblast functions including Dkk-1 as suggested by our study. The potential role of tumour-derived Dkk-1 in mediating the alterations of osteoblastic and/or osteolytic activity associated with breast cancer however remains to be investigated. Other inhibitors of the Wnt signalling pathways including Dkk-4, which has been recently shown to be expressed in primary breast tumours (Wirths *et al*, 2003; Katoh and Katoh, 2005) could also play a role in the pathogenesis of breast cancer bone metastases.

In summary, our data show that Dkk-1 is produced by human osteolytic breast cancer cells and higher circulating levels were found in women with breast cancer and bone metastases. Further studies are required to delineate the role of Dkk-1 in the physiopathological mechanisms of breast cancer-induced bone metastases.

## Figures and Tables

**Table 1:** Characteristics of the patients with breast cancer in complete remission, breast cancer and bone metastases, and breast cancer and metastases at non-bone sites

**Characteristics**	**Patients with breast cancer in complete remission**	**Patients with breast cancer and bone metastases**	**Patients with breast cancer and metastases at non-bone sites**
**No. of patients/total no. (%)**	**(*n*=17)**	**(*n*=19)**	***n*=(16)**
Age: median year (range)	58 (46–78)	66 (45–84)	52 (32–72)
			
*Histology*			
Ductal carcinoma	11/17 (65)	10/19 (52)	10/16 (63)
Lobular carcinoma	3/17 (18)	3/19 (16)	2/16 (12)
Other	0/17 (0)	3/19 (16)	0/16 (0)
Unknown	3/17 (3)	3/19 (16)	4/16 (25)
			
*Type of bone metastases*			
Lytic		5/19 (26)	
Blastic		5/19 (26)	
Mixed		5/19 (26)	
Unknown		4/19 (22)	
			
*Bulk of bone metastases*			
⩾5		12/19 (63)	
2–5		3/19 (16)	
1		3/19 (16)	
Unknown		1/19 (5)	
			
*Oestrogen receptor*			
Positive	6/17 (35)	15/19 (78)	9/16 (56)
Negative	5/17 (30)	2/19 (11)	5/16 (31)
Unknown	6/17 (35)	2/19 (11)	2/16 (13)
			
*Therapy*			
Radiation		0/19 (0)	0/16 (0)
Chemotherapy		3/19 (16)	6/16(38)
Hormonal		13/19 (68)	6/16(38)
Unknown		3/19 (16)	4/16(24)
